# Frequency of Drug Induced Acute Kidney Injury in Pediatric Intensive Care Unit

**DOI:** 10.7759/cureus.19689

**Published:** 2021-11-18

**Authors:** Murtaza A Gowa, Rabia Yamin, Hina Murtaza, Hira Nawaz, Ghazala Jamal, Pooja D Lohano

**Affiliations:** 1 Pediatrics Critical Care, National Institute of Child Health, Karachi, PAK; 2 Pediatrics, National Institute of Child Health, Karachi, PAK; 3 Pediatrics and Child Health, National Institute of Child Health, Karachi, PAK

**Keywords:** nich, pediatric intensive care unit, critically ill child, nephrotoxic drugs, drug induced aki

## Abstract

Background: Acute kidney injury (AKI) is known to complicate one-third of cases in pediatric intensive care units (PICU), and almost one-fourth of these are due to nephrotoxic drugs (NTDs). Although stopping NTDs seems the most obvious option, it is not practically applicable. Many NTDs are the only existing option, and their potential benefits outweigh the risk of drug-induced AKI.

Objectives: To assess the proportion of children receiving NTDs in the PICU and highlight the children who developed AKI.

Methods: A prospective observational study was conducted in the PICU of the National Institute of Child Health, Karachi. All children admitted to the PICU for at least 72 hours not diagnosed with any acute or chronic kidney disease were included. Serum creatinine (SCr) was done at admission and then after 72 hours. Data was entered and analyzed using IBM Corp. Released 2013. IBM SPSS Statistics for Windows, Version 22.0. Armonk, NY: IBM Corp.

Results: Of 99 children, 53 (53.5%) were male. NTD exposure was positive in 97 (97.9%), and 72 (72.7%) had high exposure (≥3 NTDs). Drug-induced AKI was diagnosed in 46 (46.5%). It was significantly related to high SCr even at admission and high NTDs exposure. The mortality rate in the AKI group was 17% compared to 4% in the non-AKI (p=0.02).

Conclusion: Almost half of all PICU admissions were infants. Almost all patients were exposed to NTDs, and three-fourth experienced high exposure. AKI developed in 46% of patients and may be predicted by raised creatinine at the time of admission. Children exposed to ≥3 NTDs had a higher chance of drug-induced AKI.

## Introduction

Acute kidney injury (AKI) may only begin as a subtle and gradual decrease in glomerular filtration rate (GFR) which may culminate in kidney failure if left to progress unchecked. It is clinically represented by azotemia and biochemically by doubling serum creatinine (SCr) levels over hours to days after the triggering event [1-3]. AKI is known to complicate 8%-30% of cases in pediatric intensive care units (PICU). Of these, almost 25% are due to pharmacotherapy [3]. According to the KDIGO (Kidney Disease: Improving Global Outcomes) definition of AKI, SCr increased by ≥0.3 mg/dL within 48 hours or by ≥1.5 times baseline within seven days with urine output less than 0.5 mL/kg/h for six hours [4].

Neonates are at a higher risk of AKI, especially those with severe asphyxia, premature birth, and who underwent cardiac surgeries [2]. Drug-induced AKI is mainly caused by antibiotics, non-steroidal anti-inflammatory drugs (NSAIDs), and chemotherapeutic agents [2]. Nephrotoxic drugs (NTDs) are not the same for children as those with adults due to differences in the renal physiology of the two. The volume of distribution, GFR, drug clearance rates, and enzymatic activation are the factors that alter pharmacokinetics in children from that in adults. The resultant systemic concentration of the drug is toxic for children and young adults [5,6].

In both critically and non-critically ill hospitalized children, AKI is known for complicating the outcome. It prolongs the hospital stay, may result in, or increase the risk of chronic kidney disease (CKD), and may even increase the risk of mortality. Children receiving NTDs, undergoing cardiac surgeries, children with sepsis, and who are admitted to the PICU are high-risk groups for developing AKI [1]. The most controllable of these risk factors are NTDs. Where cessation of NTDs seems like the most obvious way to prevent drug-induced AKI in children, it is not the most practical and clinically applicable solution. Most of the NTDs for children is also the only existing options in some life-threatening conditions, and their potential benefits outweigh the risk of drug-induced AKI. However, dose adjustment and aggressive hydration are practical efforts towards the protection of the kidneys [3,7].

There are not many local pieces of evidence on the incidence of drug-induced AKI in Pakistani children. Ishaque et al., in 2017, reported that 58% of children admitted in PICU who were exposed to NTDs developed AKI [8]. Another local study that assessed the outcome of children with AKI reported that 59% recovered, 15% developed CKD, 19% developed end-stage renal disease (ESRD) and there were 5% mortalities. Since the study was conducted in a specialized kidney hospital, it is assumed that all patients also had primary renal-related diagnoses. Drug-induced AKI was only in 2.7% of their cases [9]. In view of the existing data, we constructed this study. Its main aim is to assess the proportion of children receiving NTDs in the PICU of a tertiary care setting in a low-income country. Further, it assessed the frequency of children who developed AKI during their PICU stay regardless of their primary diagnosis. Since this study was done in a single-center, on critically ill hospitalized patients so the data can't be generalized to the general population.

## Materials and methods

This prospective observational study was conducted in the Pediatric Intensive Care Unit (PICU) of the National Institute of Child Health, Karachi. The study was conducted after ethical approval from the institutional review board. In this study, all children of age one month to 16 years admitted to the PICU for more than, at least, 72 hours were recruited. However, children of any known chronic kidney disease or AKI at the time of presentation were excluded from the study. For all included patients, SCr levels were done twice during the study, first at admission and then after 72 hours. SCr levels are routinely done at the hospital laboratory; since it is a public hospital, no amount is charged. Hence, the study did not put any additional monetary or otherwise burden on the patients or their families.

In order to collect patient information, a semi-structured questionnaire were constructed. It included demographic data such as age and gender was recorded. Clinical and biochemical characteristics included working diagnosis, all details of pharmacotherapy, serum creatinine levels, need for mechanical ventilation, and inotropic agents if used. Patients were categorized as exposed to nephrotoxic drugs if their regimen included any of the NTDs as described in Naughton et al. [10]. If patients received three or more NTDs they were termed as “High Nephrotoxic Exposure.” Acute kidney injury was defined according to Kidney Disease Improving Global Outcome (KDIGO) criteria [4].

Data was entered and analyzed using IBM Corp. Released 2013. IBM SPSS Statistics for Windows, Version 22.0. Armonk, NY: IBM Corp. For categorical variables, frequencies and percentages were calculated, and Chi-square was applied for correlation. For continuous variables, mean and standard deviation (SD) was calculated, and an independent T-test was applied for correlation. P value ≤0.05 was taken as significant.

## Results

Ninety-nine children were included in the study. There were 53 (53.5%) male and 46 (46.5%) female children. Their mean age was 41.1 ± 43.7 months (range: 2-180 months). There were 42 (42.5%) infants (1-12 months of age), 34 (34.3%) children of age 1-5 years, and 15 (15.2%) of age 5-10 years, and eight (8.1%) children of age 10 years or more. Their working diagnosis is categorized in Table 1.

**Table 1:** Working diagnosis of the children included in the study

Working diagnosis	Frequency n (%)
Acute liver failure	2 (2.0%)
Addison’s disease	1 (1.0%)
Acute gastroenteritis with sepsis	24 (24.2%)
Acute respiratory distress syndrome with pneumothorax with sepsis with multi-organ failure	5 (5.1%) 1 (1.0%) 1 (1.0%) 1 (1.0%)
Bronchogenic cyst	1 (1.0%)
Bronchopneumonia/pneumonia	7 (7.1%)
Celiac crisis	4 (4.0%)
Chemical pneumonitis	1 (1.0%)
Complicated measles	2 (2.0%)
Cystic fibrosis	1 (1.0%)
Diabetic ketoacidosis	1 (1.0%)
Encephalopathy hepatic hypertensive	2 (2.0%) 1 (1.0%) 1 (1.0%)
Encephalitis/Meningitis meningitis encephalitis meningoencephalitis meningitis with sepsis	10 (10.1%) 4 (4.0%) 1 (1.0%) 4 (4.0%) 1 (1.0%)
Gullian barre syndrome	3 (3.0%)
Hemolytic disease of newborn	2 (2.0%)
Inborn errors of metabolism	3 (3.0%)
Liver abscess	1 (1.0%)
Myocarditis	3 (3.0%)
Pneumothorax	2 (2.0%)
Salicylate poisoning	1 (1.0%)
Sepsis	2 (2.0%)
Status epilepticus	1 (1.0%)
Suspected/diagnosed malignancy	3 (3.0%)
Tuberculous meningitis	3 (3.0%)
Tetanus	10 (10.1%)
Urosepsis	1 (1.0%)
Ventricular septal defect with pneumothorax with pneumonia	2 (2.0%) 1 (1.0%) 1 (1.0%)

During the PICU stay, 97 (97.9%) children were exposed to NTDs, and 72 (72.7%) had high exposure (≥3 NTDs). Antibiotics were the most common NTDs. There were 46 (46.5%) children who were diagnosed with drug-induced AKI. Their characteristics are compared to those who did not develop AKI in Table 2. Table 2 shows that children who developed drug-induced kidney injury had their SCr already significantly raised at the time of admission (p=0.000) and also at 72 hours (p=0.000) after initiating therapy. All of the AKI patients and 96% of non-AKI patients were exposed to NTDs. NTD exposure did not hold any statistical value (p=0.18); high exposure (≥3 NTDs) was significantly related to AKI (p=0.012). Another interesting finding is that the mean duration of therapy was significantly shorted for the AKI group (p=0.048), which may be due to cessation of therapy due to AKI. There was no mortality in our study. Although more children with AKI were critical enough to require mechanical ventilation, there was no statistical difference between the two groups. The outcome was assessed in terms of mortality, which was poor in the AKI group (Table 2).

**Table 2:** Comparison of patient characteristics and outcome in AKI and non-AKI group

Variables	Acute kidney injury (n=46; 46.5%)	No Acute kidney injury (53; 53.5%)	P value
Gender			
Male	25 (54.3%)	28 (52.8%)	0.88
Female	21 (45.7%)	25 (47.2%)	
Mean age, months	42.3 ± 44.6	40.1 ± 43.3	0.80
Mean serum creatinine, mg/dL			
At admission	1.6 ± 2.2	0.4 ± 0.2	0.000
At 72 hours	2.4 ± 2.1	0.5 ± 0.3	0.000
Exposure to nephrotoxic drugs (%)			
Yes	46 (100%)	51 (96.2%)	0.18
No	0	2 (3.8%)	
High nephrotoxic exposure (%)			
Yes	39 (84.8%)	33 (62.3%)	0.012
No	7 (15.2%)	20 (37.7%)	
Mean duration of therapy with nephrotoxic drugs, days	4.2 ± 1.4	5.2 ± 3.2	0.048
Mortality			
Yes	8 (17.4%)	2 (3.8%)	0.02
No	38 (82.6%)	51 (96.2%)	
Need for mechanical ventilation (%)			
Yes	39 (84.8%)	43 (81.1%)	0.63
No	7 (15.2%)	10 (18.9%)	

## Discussion

Almost all children admitted to the PICU were exposed to NTDs, and three-fourth experienced high exposure. AKI developed in 46% of patients and may be predicted by raised creatinine at the time of admission. Children exposed to ≥3 NTDs have a higher chance of drug-induced AKI.

Exposure to NTDs drugs, especially antimicrobial agents, has long been an issue for critical care facilities. In a cohort of critically ill children administration of NTDs increased the risk of AKI by 3.37 times [11]. Renal dysfunction may occur within 24 hours in almost half of the pediatric patients who develop AKI suggesting the etiology to be external or community-acquired. Over the last two decades, there has been a transition in the primary cause of AKI in hospitalized critical patients from renal or systemic diseases to the use of NTDs [12]. In children with drug-associated AKI, the risk factors include younger age, SCr on the day of admission, higher baseline GFR, and the duration of NTD exposure. In critically ill children, cardiac comorbidity, coagulopathy, mechanical ventilation, hypoxemia, and hypotension may also increase the risk of AKI [12]. In our study, patients who developed AKI were slightly older and had significantly higher SCr at baseline. However, in Xu et al., the baseline SCr was comparable in all groups [13].

In a case-control retrospective report from PICU, 53% of patients who developed AKI were exposed to at least one or more NTDs as compared to 13% who did not develop AKI. These children had a mean longer duration of hospital stay. The odds ratio (OR) for AKI was highest with tropisetron (20.8), followed by metamizole (OR: 4.6) and paracetamol (OR: 3.5) [14]. Paracetamol has established safety and efficacy in children. There were still concerns with its renal and hepatic safety, as the literature has reported cases of liver damage and renal insufficiency [15,16]. Use NSAIDs and proton pump inhibitors have also been associated with a high incidence of AKI in critically ill children [13]. In a retrospective cohort with congenital heart disease children, ketorolac was the most commonly administered NTD. They reported AKI to be more common in children with high NTD exposure, although severe AKI was comparable between those with and without high NTD exposure [17]. Comparatively, in our study, AKI was more common in children with high NTD exposure.

There are adverse implications of drug-induced AKI in already critically ill children. It is associated with a higher rate of mortality. AKI also prolongs the duration of both ICU and non-ICU stay [13,18-20]. Some literature has also shown the consistent association of AKI with long-term sequelae, including chronic kidney disease [21,22]. The mortality rate in our study was significantly high in the AKI group, although the need for mechanical ventilation was comparable in both groups. However, the higher frequency of mortality may or may not be exclusive to AKI, and the underlying medical or surgical diagnosis also plays a crucial role.

This study is one of its kind from a low-resource setup in Pakistan. It has enabled us to understand the iatrogenic renal burden our children are under. It further creates a basis for more research in this sector studying the exact burden of the common nephrotoxic drugs, their alternatives, and long-term sequelae of AKI in these critically ill children.

## Conclusions

Almost half of all PICU admissions were infants. Almost all patients were exposed to NTDs, and three-fourth experienced high exposure. AKI developed in 46% of patients and may be predicted by raised creatinine at the time of admission. Children exposed to ≥3 NTDs had a higher chance of drug-induced AKI. PICU patients must be monitored for AKI from the time of admission. Adequate preventive measures must be taken for high-risk patients, and in case of incidence of AKI, it should be urgently managed to prevent any adverse outcome.
